# Plasma Renin: A Useful Marker for Mineralocorticoid Adjustment in Patients With Primary Adrenal Insufficiency

**DOI:** 10.1210/jendso/bvae174

**Published:** 2024-10-11

**Authors:** Cécilia Piazzola, Bleunn Dreves, Frédérique Albarel, Jérémie Nakache, Julia Morera, Michaël Joubert, Thierry Brue, Yves Reznik, Frédéric Castinetti

**Affiliations:** Department of Endocrinology, Aix Marseille Univ, INSERM, UMR1251, Marseille Medical Genetics, Institut MarMaRa, and APHM, Hôpital La Conception, 13005 Marseille, France; Department of Endocrinology and Diabetology, CHU Côte de Nacre, 14000 Caen, France; Department of Endocrinology, Aix Marseille Univ, INSERM, UMR1251, Marseille Medical Genetics, Institut MarMaRa, and APHM, Hôpital La Conception, 13005 Marseille, France; Department of Public Health and Biostatistics, Aix-Marseille University, 13005 Marseille, France; Department of Endocrinology and Diabetology, CHU Côte de Nacre, 14000 Caen, France; Department of Endocrinology and Diabetology, CHU Côte de Nacre, 14000 Caen, France; Department of Endocrinology, Aix Marseille Univ, INSERM, UMR1251, Marseille Medical Genetics, Institut MarMaRa, and APHM, Hôpital La Conception, 13005 Marseille, France; Department of Endocrinology and Diabetology, CHU Côte de Nacre, 14000 Caen, France; Department of Endocrinology, Aix Marseille Univ, INSERM, UMR1251, Marseille Medical Genetics, Institut MarMaRa, and APHM, Hôpital La Conception, 13005 Marseille, France

**Keywords:** primary adrenal insufficiency, renin, mineralocorticoids, glucocorticoids, potassium

## Abstract

**Context:**

Renin is a marker of blood volume. There is no consensus on the validity of plasma renin measurement for adjusting mineralocorticoid (MC) substitution in patients with primary adrenal insufficiency (PAI).

**Objective:**

This work aimed to investigate if plasma renin could be used to adjust MC substitution in patients with PAI.

**Methods:**

A total of 150 patients with at least one measurement of plasma renin followed for PAI at 2 tertiary expert centers between 2008 and 2022 were retrospectively included. As supraphysiological hydrocortisone might have additional MC activity, we integrated the individual hydrocortisone dose to obtain the MC equivalent dose (Eq-MC). Renin less than 20 mIU/L was considered oversubstituted, renin between 20 and 60 mIU/L as correctly substituted, and renin over 60 mIU/L as undersubstituted.

**Results:**

The mean dose of fludrocortisone was 82.3 ± 46 μg/day. Plasma renin was abnormal in 56.7% of cases (7 patients oversubstituted and 78 patients undersubstituted). Abnormalities in electrolyte levels were observed in only 12.7% of patients. Plasma renin correlated negatively with sodium (*P* < .01) and systolic blood pressure (*P* = .026), and positively with potassium (*P* < .01). Doses changes in Eq-MC had a statistically significant effect on renin levels (*P* = .0037), with an increase of MC dose correlating with a decrease in renin level and vice versa; no correlation was observed using electrolytes or blood pressure.

**Conclusion:**

Plasma renin correlates with electrolytes and blood pressure. While dose changes significantly alter renin levels, electrolytes and blood pressure do not, suggesting that renin may provide more information about MC replacement therapy than electrolytes and blood pressure.

Renin is an enzyme secreted by the juxtaglomerular cells of the kidney. It is produced after a drop in pressure in the renal artery. Other situations such as catecholamine increase or the use of diuretics or estrogens can increase renin, while age, diabetes, and chronic kidney diseases can decrease renin. Its main function is to stimulate the secretion of aldosterone through the renin-angiotensin-aldosterone system [1]. Aldosterone then acts through mineralocorticoid (MC) receptors to allow sodium reabsorption and potassium secretion to maintain blood pressure within the target range. In primary adrenal insufficiency (PAI), the lack of glucocorticoid (GC) and MC production requires an optimal replacement [2]. However, while recommendations for GC replacement are relatively well codified, with a daily dose of 8.1 mg/m^2^ per day in 3 intakes [3, 4], those for an appropriate MC replacement remain unclear.

In fact, since the discovery of fludrocortisone by Joseph Fried in 1954 [5], few studies have focused on how to optimize its use. Clinically, it is often difficult to distinguish between signs of GC and MC deficiency [2]. Endocrinologists therefore mainly focus on blood pressure and blood potassium to tailor fludrocortisone [6], with a starting dose of 50 to 100 µg/day and no salt restriction. Although renin is the most accurate marker of blood volume, there is no consensus on the use of plasma renin measurement to guide fludrocortisone dosing: Some suggest that plasma renin concentration should be in the upper normal reference range [2], but this remains controversial. Since the first study showing correlations between fludrocortisone, plasma renin activity (PRA), and systolic blood pressure (SBP) in 22 patients [7], doubts have been raised about the reproducibility of PRA, especially for extreme values. Therefore 2 studies focused on plasma renin concentration [8]. However, while Ceccato et al [9] reported a negative correlation between fludrocortisone and renin, with higher doses observed in patients with low-normal renin, Pofi et al [10] showed no correlation between renin and fludrocortisone, raising questions about the use of renin in the management of patients with PAI. Interestingly, renin may also be affected by GC replacement, an issue not considered in previous studies. MCs and GCs have analogous DNA-binding domains and similar ligand-binding domains, and therefore cortisol has a high affinity for the MC receptor. However, in target tissues, cortisol is metabolized to cortisone. Cortisol therefore does not normally have MC activity, suggesting that hydrocortisone (ie, cortisol) at physiological doses should not have MC activity either. However, a recent study using hair cortisol to assess cortisol exposure suggests that the recommended doses of hydrocortisone given in adrenal insufficiency (15-25 mg/day) may be supraphysiological [11]. This would result in an additional MC effect of hydrocortisone in adrenal insufficiency. Although controversial, it has been suggested that 1 mg of hydrocortisone is equivalent to 2.5 μg of fludrocortisone [12].

Given the lack of conclusive data in the literature, and the hypothesis that a correct interpretation of renin should consider both fludrocortisone and hydrocortisone doses, we decided to perform a bicentric retrospective study to determine if and how renin could be used in clinical practice to adjust the dose of fludrocortisone in patients with PAI.

## Materials and Methods

### Population, Clinical and Biological Data

We retrospectively included the data from patients followed up for PAI between 2008 and 2022 at 2 tertiary expert centers, namely the department of endocrinology of Assistance Publique–Hôpitaux de Marseille, and the department of endocrinology of Caen hospital. PAI was defined by low cortisol and elevated adrenocorticotropin (ACTH) with an etiologic diagnosis such as the following:

- autoimmune diseases with positive antiadrenal cortex or anti–21-hydroxylase antibodies (isolated or associated within a polyendocrine syndrome);
- bilateral adrenalectomy, mainly for pheochromocytomas;
- classic congenital adrenal hyperplasia due to 21-hydroxylase deficiency (nonclassic forms were excluded); or
- other causes of PAI, such as bilateral hemorrhage, infection (mainly tuberculosis), X-linked adrenoleukodystrophy, or mitotane-induced PAI.

Adult patients with PAI were included if at least one measurement of plasma renin concentration was available during follow-up (T0). Renin measurement was considered valid only when patients had been on a stable dose of GCs and MCs for at least 1 month at the time of this measurement. For this reason, we did not include baseline data at the time of diagnosis. If patients had multiple renin measurements during follow-up after at least 1 month on stable doses of GCs and MCs, up to 3 different renin measurements could be included (T0, T1, and T2).

Sex, cause of PAI, and renin measurement were recorded. At the same time as each renin measurement, we collected the following: dose of fludrocortisone and GC treatment (hydrocortisone and other corticoids, such as dexamethasone, prednisone, and prednisolone), weight and height, SBP and diastolic blood pressure (DBP), electrolytes, concomitant medications by antihypertensive drugs, and type of treatment. Renin assays performed on antihypertensive drugs that modify renin levels were not included. Of note, patients receiving salt supplementation were excluded.

MC equivalent (Eq-MC) calculation was performed considering that 1 mg of hydrocortisone was equivalent to 2.5 μg of fludrocortisone and that prednisone and prednisolone had 80% of the MC effect of hydrocortisone, whereas dexamethasone had no MC effect.

The majority of samples were performed at the 2 university centers, using the IDS-iSYS Direct Renin Assay on EDTA sample (reference: IS-3400). This assay uses a sandwich chemiluminescence immunoassay technique with a lower limit of detection of 1.8 mIU/L and linearity spanning from 1.8 to 550 mIU/L, with normal values from 4.2 to 59.7 mIU/L. Some measurements were performed outside these university hospitals, using chemiluminescence. Exact details of these assays were not available; however, the upper limit of the normal range was less than 46.1 mIU/L in all of them. For those values available in pg/mL, the conversion factor provided by each individual assay kit was used, that is, most commonly, pg/mL × 1.67. It should be noted that the timing of laboratory testing may vary from patient to another. Renin was considered optimal between 20 and 60 mIU/L, as suggested by the French guidelines [13], which recommend avoiding strict normalization, as this may lead to edema and hypokalemia, and suggest maintaining renin in the high normal range or slightly above. We then performed a subgroup analysis based on plasma renin concentration, in 3 groups, less than 20 mIU/L (considered oversubstituted), between 20 and 60 mIU/L (considered as correctly substituted), and greater than 60 mIU/L (considered undersubstituted). Regarding electrolytes, normal sodium was between 136 and 145 mmol/L and normal potassium was between 3.5 and 5 mmol/L.

This study was approved by the ethics committee of Assistance Publique–Hôpitaux de Marseille (RGPD PADS reference X75YVV). As per our institutional guidelines, this retrospective study did not require specific signed informed consent from patients because the data collected were anonymized.

### Statistical Analysis

The description of the study population is presented as numbers and proportions (percentages) for the qualitative variables and means ± SD for the quantitative variables. Then, the correlation between quantitative variables was studied using the nonparametric Spearman test.

The 3 main etiologies were compared using the Kruskal-Wallis test. The variables of interest were compared between the 3 renin groups using ordinal logistic regression. The estimated parameters are presented with their 95% CIs.

The analysis was performed using IBM SPSS Statistics version 20 software.

## Results

A total of 150 patients (49 males, 32.7% and 101 females, 67.3%, with a mean age at diagnosis of 29 ± 7.8 years) were included in this study. As shown in Table 1, the majority presented with autoimmune disease (37.3%), while 30.6% had undergone bilateral adrenalectomy for pheochromocytoma and 24.6% had classic congenital adrenal hyperplasia. A total of 143 patients (95%) were treated with hydrocortisone at a mean dose of 25.2 ± 10 mg/day (minimum 7.5 mg/day, maximum 60 mg/day), while 138 (92%) were treated with fludrocortisone at a mean dose of 82.3 ± 46 μg/day (minimum 0 μg/day, maximum 250 μg/day). When considering the GC dose, the mean equivalent MC dose (with, as explained earlier, 1 mg of hydrocortisone equivalent to 2.5 μg of fludrocortisone) was 135.9 ± 51 μg per day. Interestingly, 14 patients were treated with antihypertensive drugs, 7 with therapies considered neutral toward the renin-angiotensin system (mainly calcium antagonists) and 7 with renin-angiotensin system inhibitors (excluded in the future analysis): Two of these patients did not receive fludrocortisone.

**Table 1.** Characteristics of the population (mean ± SD or number, N, and percentage)

	T0	T1	T2
No. of patients	150	106	53
Women/Men (N/ %)	101/49 (67.3/32.7%)	77/29 (72.6/27.4%)	38/15 (71.7/28.3%)
Age, y	45.1 ± 18.9	44.4 ± 18.2	46.2 ± 19
BMI	25.7 ± 5.4	25.8 ± 6.1	26.5 ± 5.3
Causes, N			
Autoimmune	56 (37.3%)	38 (35.8%)	18 (34%)
Bilateral adrenalectomy	46 (30.6%)	27 (25.5)	14 (26.4%)
21-Hydroxylase deficiency	37 (24.6%)	35 (33%)	20 (37.8%)
Other*^a^*	11 (7.3%)	6 (5.7%)	1 (1.9%)
Hydrocortisone			
No. of patients	143 (95%)	99 (93.4%)	47 (88.7%)
Mean dose, mg	25.2 ± 10.3	24 ± 11	24.6 ± 11.8
Dexamethasone			
No. of patients	7 (4.6%)	6 (5.7%)	5 (9.4%)
Mean dose, mg	0.43 ± 0.1	0.45 ± 0.1	0.45 ± 0.1
Prednisone			
No. of patients	4 (2.6%)	3 (2.8%)	2 (3.8%)
Mean dose, mg	5.5 ± 1.1	5.5 ± 1.7	6 ± 1.2
Prednisolone			
No. of patients	0	1 (0.9%)	1 (1.9%)
Mean dose, mg		8 ± 0.8	8 ± 1.1
Fludrocortisone			
No. of patients	138 (92%)	100 (94.3%)	50 (94.3%)
Mean dose, µg	82.3 ± 46.9	95 ± 52	101.5 ± 49.7
Equivalent MC, µg	135.9 ± 51.5	145.9 ± 53	150.9 ± 54.2
Renin, mIU/L	121.7 ± 173.7	102.2 ± 176.8	134.8 ± 539.6
Sodium, mmol/L	139.1 ± 2.8	139.7 ± 2.5	139.9 ± 2.7
Potassium, mmol/L	4.2 ± 0.4	4.2 ± 0.4	4.1 ± 0.4
SBP, mm Hg	120.4 ± 14.4	124.1 ± 17.8	122.8 ± 15
DBP, mm Hg	73 ± 10.6	71.7 ± 10.2	71 ± 11.8
Antihypertensive therapy, N	14 (9.3%)	15 (14.2%)	7 (13.2%)
Monotherapy	10	11	5
Bitherapy	3	2	1
Tritherapy	1	2	1

Abbreviations: BMI, body mass index; DBP, diastolic blood pressure; MC, mineralocorticoid; SBP, systolic blood pressure.

^*a*^Tuberculosis: 3/11 patients, lymphoma: 1/11, mitotane-induced: 1/11, immunotherapy-induced: 1/11, genetic (NNT mutation, Allgrove syndrome, X-linked adrenoleukodystrophy): 4/11, unknown: 1/11.

Finally, of the 150 patients included, 106 had at least 2 renin measurements during follow-up (mean time from T0 to T1 was 1 ± 21 months) and 53 had 3 renin measurements available (mean time from T1 to T2 was 23 ± 19 months) (see Table 1). Mean renin at T0 was 121.7 ± 137 mIU/L (and median was 48 mIU/L). Renin was considered abnormal by laboratory standards in 56.7% of cases, with 7 patients (4.7%) below the lower limit of normal and 67 patients (44.7%) above the upper range. Regarding electrolytes, 131 patients (87.7%) had normal sodium and potassium. Thus, only 12.7% of our patients had hyponatremia and/or hyperkalemia or even hypokalemia. Eleven of 150 patients (7.3%) had hyponatremia at T0 (mean renin 363.9 ± 194.8 mIU/L), while 6 patients had hyperkalemia (mean renin 408.6 ± 420.2 mIU/L). On the other hand, 3 patients had hypokalemia (mean renin 19.4 ± 2.8 mIU/L), but none had hypernatremia. It is important to note that all patients with hyponatremia and/or hyperkalemia had very high renin levels and none of them had renin levels in the normal range. The comparison between the 3 main groups (autoimmune disease, bilateral adrenalectomy, and 21-hydroxylase deficiency) is shown in Table 2. Patients with 21-hydroxylase deficiency were younger than the others. In addition, patients with bilateral adrenalectomy received lower doses of fludrocortisone, but this was offset by higher doses of GCs (nonsignificantly), and thus a dose of Eq-MC comparable to that of the other groups.

**Table 2.** Characteristics of the 3 main groups at T0 (mean ± SD or number, N, and percentage)

Parameters	Autoimmune	Bilateral adrenalectomy	21-Hydroxylase deficiency	*P*
No. of patients	55	46	37	/
Women/Men (N/%)	42/13 (76.3/23.6)	32/14 (69.6/30.4)	23/14 (62.2/37.8)	.34
Age, y	48.3 ± 16.7	53.1 ± 14.9	26.1 ± 10.7	<.001
BMI	24.2 ± 3.4	27.3 ± 4.6	24.6 ± 6.5	.003
Hydrocortisone				
No. of patients	54	45	32	.16
Mean dose, mg	23.5 ± 7.3	27.2 ± 9.2	26.2 ± 11.6	
Dexamethasone				
No. of patients	0	0	7	<.001
Mean dose, mg			0.43 ± 0.17	
Prednisone				
No. of patients	1	1	2	.57
Mean dose, mg	5	5	6 ± 7.1	
Prednisolone				
No. of patients	0	0	0	NS
Mean dose, mg				
Fludrocortisone				
No. of patients	51	41	35	.036
Mean dose, μg	82.4 ± 38.7	74.7 ± 43.7	97.6 ± 46.5	
Equivalent MC, μg	134.3 ± 47.9	133.2 ± 49.3	149.2 ± 61.1	.26
Renin, mIU/L	158 ± 194.6	124.5 ± 204.5	82.1 ± 95.3	.4
Sodium, mmol/L	138.3 ± 3.1	139.4 ± 2.7	139.5 ± 2.4	.14
Potassium, mmol/L	4.2 ± 0.4	4.3 ± 0.5	4.1 ± 0.4	.29
SBP, mm Hg	118.6 ± 14.5	120.5 ± 15.8	121 ± 13	.7
DBP, mm Hg	73.4 ± 10.6	73.6 ± 11.3	71.8 ± 11.2	.86
Antihypertensive therapy, N	2	8	0	.003
Monotherapy	1	6		
Bitherapy	0	2		
Tritherapy	1	0		

Abbreviations: BMI, body mass index; DBP, diastolic blood pressure; MC, mineralocorticoid; NS, not significant; SBP, systolic blood pressure.

First, we analyzed the correlation between renin and other parameters at T0 (Table 3).

**Table 3.** Correlation between renin and other parameters at T0

Correlation renin and	*P*	rho
Age	.287	0.088
BMI	.359	0.0898
Sodium	<.01	−0.420
Potassium	<.01	0.393
SBP	.026	−0.2
DBP	.792	−0.024
Hydrocortisone	.285	0.088
Fludrocortisone	.829	0.018
Eq MC	.462	0.061

Abbreviations: BMI, body mass index; DBP, diastolic blood pressure; Eq, equivalent; MC, mineralocorticoid; SBP, systolic blood pressure.

Results were statistically significant if *P* was less than .05.

**Figure 1.**
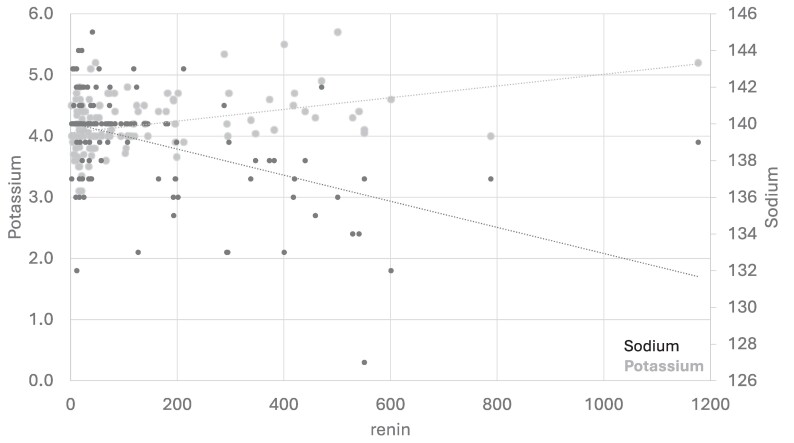
Correlation between sodium, potassium, and renin (linear regression) at baseline.

Renin levels were negatively correlated both with sodium (*P* < .01) and SBP (*P* = .026), but positively correlated with potassium (*P* < .01) (Fig. 1). No correlation was observed between plasma renin concentration and fludrocortisone (*P* = .829) or hydrocortisone (*P* = .285). Furthermore, we observed no correlation between plasma renin and Eq-MC (*P* = .462; *r* = 0.061), but a negative correlation between Eq-MC and age (*P* = .02; *r* = −0.189). There was a trend between Eq-MC dose and disease duration in patients with autoimmune disease, with higher doses found in patients with longer disease duration (*P* = .13; *r* = 0.233), the lack of statistical significance being possibly due to the small number of data available in this case (44 patients). No correlation was observed between Eq-MC and electrolytes or blood pressure (data not shown).

We then evaluated the characteristics of the patients at the first valid renin monitoring (T0) according to the renin rate, which was divided into 3 categories (Table 4) using ordinal regression: less than 20 mIU/L (overdosed, n = 39 patients), more than 60 mIU/L (underdosed, n = 67 patients), and between 20 and 60 mIU/L (correctly substituted, n = 44 patients). Our results showed that sodium was significantly lower (*P* < .0001) and SBP (*P* = .03) and potassium were significantly higher (*P* < .0001) in patients with renin greater than 60 mIU/L compared to patients with lower renin. We performed a multivariate ordinal logistic regression including the main variables (age, sex, and body mass index [BMI]) and variables with a *P* value less than .05 and found a difference between groups for natremia (*P* = .002) and SBP (*P* = .01), but not for kalemia (*P* = .054). These results were consistent with those reported in Table 1, which showed a correlation between electrolytes and plasma renin.

**Table 4.** Characteristics according to rate of renin at baseline using ordinal logistic regression (mean, SD, logistic regression coefficient, and CIs)

	<20 mIU/L	20-60 mIU/L	>60 mIU/L	Logistic regression coefficient (β)	95% CI	*P*
No. of patients	39	44	67		—	—
BMI	25.8 ± 4.8	24.4 ± 4.8	26.4 ± 6	0.028	(−0.035 to 0.090)	.381
Hydrocortisone, mg	23.2 ± 8.5	23.2 ± 8.8	25.1 ± 12	0.016	(−0.014 to 0.045)	.289
Fludrocortisone, μg	76.5 ± 42.2	72.2 ± 41.3	77.5 ± 52.9	0.001	(−0.006 to 0.007)	.807
Eq MC, μg	134.8 ± 39.9	130.3 ± 45.9	140.2 ± 60.4	0.002	(−0.004 to 0.008)	.478
Sodium, mmol/L	140 ± 2.5	139.9 ± 2	137.9 ± 3.1	−0.273	(−0.418 to −0.129)	<.0001
Potassium, mmol/L	4 ± 0.4	4.1 ± 0.4	4.3 ± 0.4	1.6	(0.727 to 2.474)	<.0001
SBP, mm Hg	125.5 ± 10.5	120.2 ± 15.3	117.7 ± 15.2	−0.026	(−0.05 to −0.002)	.03
DBP, mm Hg	73.9 ± 8.4	72.1 ± 8.7	78.9 ± 12.9	−0.004	(−0.036 to 0.27)	.781

Abbreviations: BMI, body mass index; DBP, diastolic blood pressure; Eq, equivalent; MC, mineralocorticoid; SBP, systolic blood pressure.

Results were statistically significant if *P* was less than .05.

**Figure 2.**
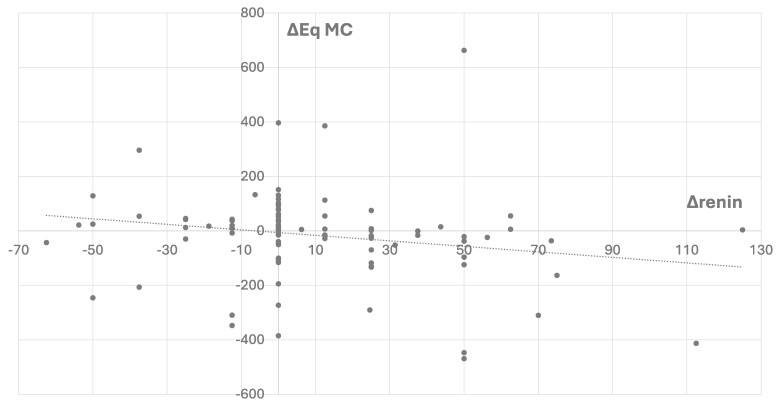
Correlation between ΔreninT1T2 and ΔEqMCT1T2 (*P* = .0037). Equation: Δrenin = −1011*(ΔEq MC) – 6105. SE = 0.48.

We then analyzed renin levels at T0-T1-T2 to determine whether dose changes in Eq-MC correlated with changes in the rate of plasma renin (Fig. 2). The increase in MC dose was significantly correlated with a decrease in renin and vice versa (*P* = .0037). However, the same analysis using electrolytes instead of renin showed no correlation, whether sodium (*P* = .21) or potassium (0.208) was used. Similarly, variations in arterial blood pressure were not significantly correlated with variations in treatment (*P* = .215).

**Figure 3.**
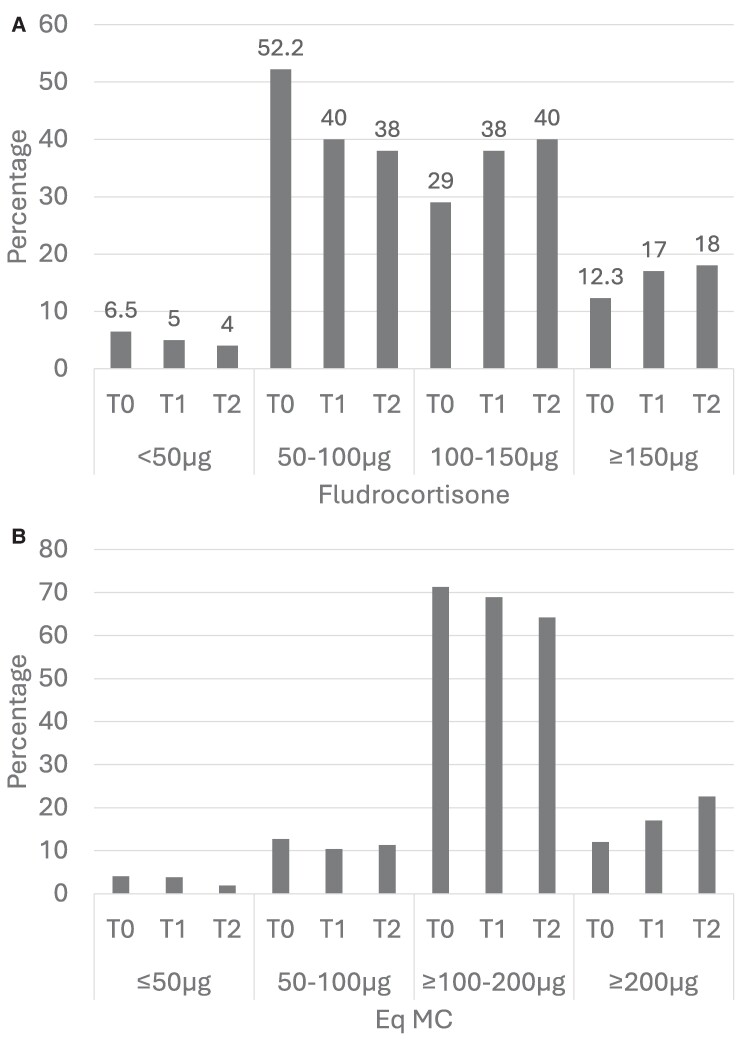
Evolution of dose over time (percentage of patients within thresholds). A, Fludrocortisone dose; B, mineralocorticoid equivalent (Eq-Mc) dose. Eq-MC calculation was performed considering that 1 mg of hydrocortisone was equivalent to 2.5 μg of fludrocortisone, that prednisone and prednisolone had 80% of the MC effect of hydrocortisone, whereas dexamethasone had no MC effect.

Interestingly, while hydrocortisone dose did not significantly change between T0 and T2 (mean 25.2 mg ± 10.3 at T0 vs 24.6 ± 11.8 mg at T2; *P* = .7), fludrocortisone dose increased during follow-up, with 58.7% vs 42% of patients below 100 μg per day at T0 and T2, respectively (*P* = .0021). The same trend was observed for Eq-MC with *P* = .05 (Fig. 3A and 3B).

Finally, we compared the evolution of renin in patients whose treatment was changed between T0 and T1, and then between T1 and T2, with that of patients on a stable dose of fludrocortisone. Our hypothesis was that renin would remain stable (ie, more or less 20% compared to baseline) in patients with a stable dose, and that it would be significantly modified in the group of patients with a variable dose. However, although renin remained stable in patients whose treatment had not changed compared with patients in whom Eq-MC was decreased (*P* = .036), no statistically significant difference was observed with patients in whom Eq-MC had increased (*P* = .6).

## Discussion

Our results show that plasma renin concentration is a more sensitive marker than electrolytes and blood pressure for detecting over or under MC replacement dosing. Furthermore, plasma renin correlated with sodium, potassium, and SBP, with a decrease both in sodium and blood pressure and an increase in potassium when renin was greater than 60 mIU/L.

It is important to emphasize that only 12.7% of our patients had hyponatremia and/or hyperkalemia or even hypokalemia, whereas renin was considered abnormal in 46.7% of cases according to laboratory standards, and in 70.7% of cases according to our categories, confirming that electrolytes represent poorly sensitive markers. Furthermore, all patients with hyponatremia and/or hyperkalemia had very high renin levels. This suggests that only very poorly controlled patients on MC substitution receive a biological effect on electrolytes, proving that an additional marker is needed to tailor MC supplementation, and that plasma renin appears to be a more sensitive parameter than electrolytes.

In contrast, we found no correlation between plasma renin and fludrocortisone dose. These results are consistent with those of Pofi et al [10], although they did not show a significant difference in renin levels as a function of treatment variations. This could be due to differences in their population, with the majority of their patients having congenital adrenal hyperplasia. In addition, the data were collected over a long period of time using variable assay techniques, including PRA, which is less reliable, as mentioned earlier. Regarding follow-up data, their longitudinal analysis was performed on 112 adults, but only 33% had their treatment changed, which may have led to a lack of power and explain our discrepancy on this point, since on our side, the treatment had been changed in 53% of cases. In contrast, Ceccato et al [9] found a positive correlation between fludrocortisone dose and sodium levels and a negative correlation with potassium and plasma renin concentration, in a single-center study with a larger number of patients. The population was similar to ours, but the patients had higher doses of GC substitution (32 mg/day initially) and lower doses of fludrocortisone than in our study, which may explain some of the differences between both results.

Since supraphysiological GC substitution may alter MC activity, we hypothesized that renin may correlate with a combined index that considers both GC and MC doses (Eq-MC) rather than MC dose alone. Interestingly, changing the dose of Eq-MC significantly altered renin levels, suggesting that both parameters should be considered when interpreting renin. We did not find any correlation between the change in Eq-MC and electrolytes or even blood pressure, suggesting that these parameters are not sufficient to adjust MC replacement. We believe that these results are probably due to the small number of patients with electrolyte abnormalities, as mentioned earlier. Interestingly, we found no correlation between Eq-MC and renin measurements. This could be explained by the interindividual variability in MC requirements: For a given dose of fludrocortisone, one should not expect the same renin changes in each patient. This could be due to differences in the patients’ 11β-hydroxysteroid dehydrogenase type 1 and 2. This underscores the importance of our Eq-MC calculation, which, although potentially questionable, is of interest in cases of hydrocortisone overdose. Moreover, in line with our finding of a decreasing dose of MC with age, there are differences in renal tubule maturation with age, with plasma renin concentration lower in older than in younger patients, as shown in the study by Weidmann et al [14], as well as a trend toward a reduction in aldosterone consistent with our results, finding lower doses of MCs in older patients. In addition, reduced renin secretion is often observed in patients with sclerosis of the juxtaglomerular apparatus or renal vascular changes, but none of our patients had severe or end-stage renal disease. It is also interesting to note that one-third of patients still produce GC after onset of PAI, even decades after diagnosis, and 1 in 7 still produce MC, with lower doses of fludrocortisone in patients with shorter median disease duration as shown in the study by Sævik et al [15]. It is also possible that patients have different absorption rates and drug metabolism patterns. All these parameters could thus modify the renin response to MC supplementation, making the use of renin a good marker for fludrocortisone supplementation rather at an individual than at a generalized level. Furthermore, an optimal dose of MC supplementation appears to be necessary for cardiovascular function: It is now well established that hyperaldosteronism is an independent cardiovascular risk factor, with an increased incidence of stroke, coronary heart disease, atrial fibrillation, and heart failure in patients with hyperaldosteronism compared with essential hypertension [16]. Underlying mechanisms appear to be impaired endothelial function independent of the effect of blood pressure on cardiovascular disease progression [17]. Patients with overreplacement in MC substitution may have cardiovascular disease like those with hyperaldosteronism, as shown in preliminary studies [18], but may also have defects in immune cell signaling, adipocyte differentiation, and central nervous system function [19]: A recent study showed that correct substitution is necessary for cognitive function [20], especially verbal memory and mood. Of course, it is difficult to confirm overdosing or underdosing as there is really no “gold-standard’ marker of correct MC replacement. However, given all the possible side effects described previously, it seems clear that optimal MC replacement is needed and that the clinician cannot rely on electrolytes alone. Therefore, although imperfect, renin may be a good marker to evaluate MC replacement from an individual perspective.

A major limitation of this study is its retrospective nature in a setting where there are no formal guidelines for MC dose adjustment. In addition, follow-up visits were thus performed at different time points, but we tried to minimize this bias by ensuring that there was at least 1 month between 2 measurements. Treatment adherence could not be assessed, although it could influence renin measurements, especially in patients with multiple daily intakes: In a recent study, 32% of 41 patients with adrenal insufficiency were considered to be noncompliant [21]. The frequency of hydrocortisone administration may also influence plasma renin levels; however, all of our patients were treated with similar protocols, receiving 2 or 3 doses per day, so we did not consider this point as a bias. In addition, the renin measurements were not standardized: In the majority of patients, the 2 main measurement methods used, although reporting quite similar normal values, were not exactly the same, and therefore subtle variations in renin measurements could be due only to differences in the measurement methods. In addition, some patients were assessed with other renin assays, and although the reference ranges were quite similar, this could lead to a bias in the interpretation of the results. Nevertheless, we would like to point out that all patients with hyponatremia and/or hyperkalemia had very high renin levels (well above the accepted standards), which allows us to exclude interlaboratory differences on this point. Furthermore, we had no information on posture, plasma volume variability, or daily sodium intake at the time of measurement, which could influence renin value. Regarding this last point, the management of sodium absorption in patients is quite challenging, even in a prospective study, due to poor patient adherence to dietary advice. One solution might be to monitor sodium intake by urinary sodium excretion, but this was not available in our retrospective study. The exact time of renin measurement was not known, but this does not seem to be critical as renin concentration is stable over 24 hours in patients with adrenal insufficiency [22]. Finally, it should be noted that it is difficult to exclude an effect of 11β-hydroxysteroid dehydrogenase type 2, which physiologically inactivates cortisol to cortisone; however, we estimated that this effect was minimal at the doses of hydrocortisone used.

In conclusion, our study suggests that plasma renin is a more accurate marker than electrolytes or blood pressure to detect overdosing or underdosing of MC replacement therapy. Our data suggest the inclusion of plasma renin measurement according to a standardized procedure to more accurately monitor the MC replacement dose. Our study also illustrates the wide variability of hydrocortisone replacement regimens, which is a major issue in defining optimal MC replacement. Based on our current knowledge of adrenal insufficiency, we believe that MC replacement should take into account renin, cortisol, ACTH, sodium, potassium, and blood pressure, as well as any individual characteristics (such as other treatments or comorbidities) that may affect these measures. Future prospective studies on a larger number of patients will also help determine the potential side effects of MC overdosing or underdosing, with a target zone that remains to be refined but seems to be around between 20 and 60 mIU/L.

## Data Availability

Restrictions apply to the availability of all data analyzed during this study to preserve patient confidentiality. The corresponding author will on request detail the restrictions and any conditions under which access to some data may be provided.

## Abbreviations

ACTH: adrenocorticotropin
BMI: body mass index
DBP: diastolic blood pressure
MC: mineralocorticoid
PAI: primary adrenal insufficiency
PRA: plasma renin activity
SBP: systolic blood pressure
